# One-step *in vivo* gene knock-out in porcine embryos using recombinant adeno-associated viruses

**DOI:** 10.3389/fcell.2024.1376936

**Published:** 2024-03-15

**Authors:** Mengyu Gao, YuTing He, XingLong Zhu, WanLiu Peng, YanYan Zhou, Yang Deng, Guangneng Liao, Wei Ni, Yi Li, Jun Gao, Hong Bu, Jiayin Yang, Guang Yang, Yang Yang, Ji Bao

**Affiliations:** ^1^ Department of Pathology, Institute of Clinical Pathology, Key Laboratory of Transplant Engineering and Immunology, West China Hospital, Sichuan University, Chengdu, China; ^2^ Experimental Animal Center, West China Hospital, Sichuan University, Chengdu, China; ^3^ Security Department, West China Hospital, Sichuan University, Chengdu, China; ^4^ Institute of Respiratory Health, West China Hospital, Sichuan University, Chengdu, China; ^5^ Department of Toxicological Inspection, Sichuan Center for Disease Prevention and Control, Chengdu, China; ^6^ Laboratory of Liver Transplantation, Frontiers Science Center for Disease-related Molecular, Network, West China Hospital, Sichuan University, Chengdu, China; ^7^ State Key Laboratory of Biotherapy and Cancer Center, West China Hospital, Sichuan University, Chengdu, China

**Keywords:** CRISPR/Cas9, recombinant adeno-associated viruses, embryo gene delivery, gene-edited pig, animal model

## Abstract

**Introduction:** Gene-edited pigs have become prominent models for studying human disease mechanisms, gene therapy, and xenotransplantation. CRISPR (clustered regularly interspaced short palindromic repeats)/CRISPR-associated 9 (CRISPR/Cas9) technology is a widely employed tool for generating gene-edited pigs. Nevertheless, delivering CRISPR/Cas9 to pre-implantation embryos has traditionally posed challenges due to its reliance on intricate micromanipulation equipment and specialized techniques, resulting in high costs and time-consuming procedures. This study aims to introduce a novel one-step approach for generating genetically modified pigs by transducing CRISPR/Cas9 components into pre-implantation porcine embryos through oviductal injection of recombinant adeno-associated viruses (rAAV).

**Methods:** We first used rAAV-1, rAAV-6, rAAV-8, rAAV-9 expressing EGFP to screen for rAAV serotypes that efficiently target porcine embryos, and then, to achieve efficient expression of CRISPR/Cas9 *in vivo* for a short period, we packaged sgRNAs targeting the GHR genes to self-complementary adeno-associated virus (scAAV), and Cas9 proteins to single-stranded adeno-associated virus (ssAAV). The efficiency of porcine embryos -based editing was then validated *in vitro*. The feasibility of this one-step method to produce gene-edited pigs using rAAV-CRISPR/Cas9 oviductal injection into sows within 24 h of conception was then validated.

**Results:** Our research firstly establishes the efficient delivery of CRISPR/Cas9 to pig zygotes, both *in vivo* and *in vitro*, using rAAV6. Successful gene editing in pigs was achieved through oviductal injection of rAAV-CRISPR/Cas9.

**Conclusion:** This method circumvents the intricate procedures involved in *in vitro* embryo manipulation and embryo transfers, providing a straightforward and cost-effective approach for the production of gene-edited pigs.

## 1 Introduction

Pigs, due to their anatomical, physiological, and metabolic similarities to humans, as well as their shorter growth cycles, higher reproductive rates, and lower feeding costs compared to nonhuman primates, have become prominent models for studying human disease mechanisms and gene therapy (Hein et al., 2020; Gao et al., 2023). In 2021, gene-edited pigs were instrumental in significant advancements in pig-human heart and kidney xenotransplantation, pointing toward broader utilization of gene-edited pigs in xenotransplantation research. (Griffith et al., 2022; Montgomery et al., 2022).

Currently, the predominant approach for generating gene-edited pigs involves using clustered regularly interspaced short palindromic repeat (CRISPR)/CRISPR-associated 9 (CRISPR/Cas9) in conjunction with somatic cell nuclear transfer (SCNT) or embryo microinjection. Nevertheless, these methods are contingent upon utilizing intricate micromanipulation equipment and specialized techniques. Even with the option of electroporating CRISPR/Cas9 into embryos to generate genetically modified animal models, the use of electroporation equipment remains a necessity (Tanihara et al., 2016). Consequently, the delivery of CRISPR reagents using these approaches is labor-intensive, resource-demanding, and time-consuming, resulting in substantial expenses and turnaround times.

Recombinant adeno-associated virus (rAAV) has garnered attention in the field of gene therapy due to its non-integrative nature within the host genome, low immunogenicity, and minimal toxicity. Several studies have demonstrated successful delivery of the rAAV-CRISPR system to achieve functional restoration in various target tissues, including the eye (Ruan et al., 2017; Yu et al., 2017), liver (Yin et al., 2016), heart (Carroll et al., 2016; Xie et al., 2016), lung (Platt et al., 2014), and others. As a result, rAAV has become a widely adopted viral vector for *in vivo* CRISPR/Cas9 delivery in gene therapy and the preparation of animal models (Wang et al., 2019a; Nidetz et al., 2020).

A recent investigation further underscores the safety of rAAV by demonstrating its capacity to traverse the mouse embryonic zona pellucida, a feat unattainable by lentiviral and adenoviral vectors with lower safety profiles (Yoon et al., 2018). This suggests a secure avenue for delivering the CRISPR/Cas9 system to embryos without necessitating zona pellucida removal.

The transfection efficiency of rAAV relies on molecular interactions between the viral capsid and target cell surface receptors, resulting in tissue and cell specificity (Nonnenmacher and Weber, 2012). Prior to transfecting tissues and cells, specific rAAV serotypes must be screened. Among the tested serotypes, rAAV6 exhibited efficient transfection of mouse embryos when utilized for CRISPR/Cas9-mediated *in vitro* gene editing. Subsequently, these edited embryos were transferred to surrogate mothers, leading to the successful generation of biallelic mutant mice (Yoon et al., 2018). In a separate investigation, researchers reported successful gene editing of crab monkey embryos through *in vitro* co-incubation with rAAV6-CRISPR/Cas9, highlighting the strong specificity of serotype 6 rAAV for monkey embryos (Wang et al., 2019b). Additionally, strong transfection specificity of serotype 1 and serotype 6 rAAV to rat embryos was confirmed, leading to gene knockout and knock-in *in vitro* embryo experiments through co-incubation with rAAV (Mizuno et al., 2018; Romeo et al., 2020). While many studies have demonstrated the feasibility of rAAV-mediated delivery of CRISPR/Cas9 to embryos *in vitro*, none have explored the *in vivo* delivery of rAAV-CRISPR/Cas9 to large animal embryos, and no studies have investigated the potential of rAAV to transfect porcine fertilized eggs.

Given the research advances mentioned above, we contemplate the feasibility of achieving one-step delivery of CRISPR/Cas9 to porcine fertilized eggs through oviductal injection of rAAV, aimed at generating gene-edited pigs. This approach offers the advantage of circumventing intricate *in vitro* procedures, reducing the risk of embryonic abnormalities, miscarriages, and malformations associated with *in vitro* manipulations, thereby streamlining the production of large animal models.

In this study, we seek to evaluate rAAV’s capacity to penetrate the zona pellucida of porcine embryos and explore innovative methods for *in vivo* rAAV-mediated gene editing in pigs. Our focus centers on the growth hormone receptor (GHR) gene, the disruption of which results in Laron syndrome, characterized by reduced stature. This non-lethal GHR gene phenotype offers an observable model for investigating the feasibility of utilizing the rAAV-mediated CRISPR/Cas9 system for gene editing in porcine embryos, both *in vitro* and *in vivo*.

## 2 Materials and methods

### 2.1 Animals and ethics statement

The Bama pigs used in this study were purchased from Xenomed (Chengdu, China) and bred at the Laboratory Animal Centre of West China Hospital of Sichuan University. The ethics committee of Sichuan University for Animal Research accepted this work, and all animal care and experiments followed the rules of the university’s Animal Experiment Center.

### 2.2 Oocyte collection and *in vitro* maturation

Porcine embryos used in the *in vitro* experiments were obtained via somatic cell nuclear transfer. Porcine ovaries were sourced from a local slaughterhouse, and oocytes with consistent cytoplasm and two to three layers of compact cumulus were selected for *in vitro* maturation. These cumulus-oocyte complexes (COCs) were cultured in a maturation medium, with mineral oil covering the surface, within a humidified incubator set at 38.5°C with 5% CO_2_. Following a 20-hour maturation period, the COCs were subsequently transferred for an additional 22 h to a maturation medium devoid of hormones. Oocytes displaying a distinct first polar body were then utilized for somatic cell nuclear transfer (SCNT).

### 2.3 Somatic cell nuclear transfer and embryo culture

Porcine primary kidney fibroblasts were isolated from the newborn Bama piglets as the nuclear donor as described in the previous method (Gao et al., 2022). The perivitelline space of enucleated oocytes was then injected with a solitary fibroblast donor cell. Following that, reconstructed embryos were fused and stimulated by an electric pulse (BTX, two DC pulses of 1.1 kV/cm for 60 ls) and cultured in porcine zygote medium 3 (PZM3) at 39°C and 5% CO_2_ (Zhou et al., 2015).

### 2.4 sgRNA screening for target gene

We employed the online software (http://crispor.tefor.net/)for the design of sgRNAs that targeted exon 3 of the *GHR* gene. Subsequently, we identified and selected three specific sgRNA sequences, which were cloned into the PX458 vector. These constructs were then transfected into porcine kidney fibroblasts (Gao et al., 2022), and cultured with 10% fetal bovine serum (Royacel, China). Genomic DNA was extracted from the cells 7 days post-transfection (Qiagen, Germany), and the target sequences were amplified and forwarded to PCR for Sanger sequencing using specific primers (Fw 5′-3′CAC​AAT​GGT​TTG​TCC​CTG; Rv 3′-5′ ATC​ATT​TCC​GTT​CCT​ACT). The PCR conditions were as follows, 94°C for 5 min; 94°C for 30 s, 58°C for 30 s, and 72°C for 1 min for 35 cycles; 72°C for 5 min; and a hold at 4°C. To perform the PCR, the TaKaRaTaq Hot Start Version was used (TaKaRa, Japan). Subsequently, the PCR products were sent to Tsingke Bio Company for sequencing. The sequencing data were subsequently analyzed using the online software TIDE (http://tide.nki.nl/) to assess the efficiency of the sgRNAs.

### 2.5 Transduction of pre-implantation embryos *in vitro* with AAV

Zygotes were incubated in drops of PZM3 containing the following rAAV vectors for 5–6h, scAAV1-CMV-EGFP, scAAV6-CMV-EGFP, scAAV8-CMV-EGFP and scAAV1-CMV-EGFP (Pack gene, China) at a viral load of 5 × 10^9^ GC to analyze the effect of multiple rAAV serotypes for transfection of porcine embryos; scAAV6-CMV-EGFP at 5 × 10^7^ GC, 5 × 10^9^ GC, 5 × 10^10^ GC, and 5 × 10^11^ GC to screen the optimal virus transfection dose; rAAV6-CRISPR/Cas9 target gene vectors carrying an EGFP-tagged were used to test the efficiency of rAAV-CRISPR/Cas9-mediated *in vitro* embryonic gene editing (scAAV6-U6-sgGHR-CMV-EGFP and ssAAV6-U1a-spCas9, purchased from Pack gene, at 5 × 10^10^ GC); Drops were inserted into 35 mm plates, which were submerged in mineral oil (Sigma, M8410) at 37 °C in a tissue culture incubator with 5% CO_2_ and 5% O_2_. When the incubation period was up, the embryos were transferred to new PZM3 for additional culture. To reach the stage of a compacted blastocyst, zygotes were grown for 6 days. And the expression of green fluorescent protein (GFP) was continuously observed by fluorescence microscopy.

### 2.6 Oviductal injection of rAAV-CRISPR/Cas9 to produce gene-edited pigs

To assess the effectiveness of rAAV oviductal injection for *in vivo* delivery of CRISPR/Cas9 to fertilized eggs, a 1:1 mixture of scAAV6-U6-sg*GHR*-CMV-EGFP and ssAAV6-U1a -spCas9 (5 × 10^10^ GCs each) was injected into the ampulla of the oviduct after the females had spontaneously mated within 24 h. After 30 days, the sows were ultrasonically evaluated for pregnancy sac.

### 2.7 Genotyping of GHR gene knockout pigs

At approximately 114 days, the piglets were delivered naturally. The genomic DNA of the ear and organ tissue was extracted (Qiagen, Germany), and the samples were used for PCR detection as described above.

### 2.8 H&E staining and immunohistochemistry

The wide type (WT) and *GHR* gene-edited tissue samples were fixed in 4% paraformaldehyde and embedded in paraffin. Hematoxylin and eosin (H&E) was used to stain the paraffin slices (4 μm thick) in order to see the histological changes. To detect the expression of *GHR* proteins, the organ tissues were subjected to immunohistochemistry (IHC). The IHC were performed with primary antibodies against *GHR* (1:100, no.20713-1-AP, proteintech, China). The IHC slides were scanned by a digital pathology apparatus (NanoZoomer 2.0T; Hamamatsu, Japan).

### 2.9 Western blotting

Liver tissue samples from both transgenic and wild-type pigs were subjected to total protein extraction, utilizing a protein extraction kit (Beyotime, China). Following protein extraction, the samples were combined with loading buffer and separated on 10% acrylamide polyacrylamide tris-glycine gels. The proteins were subsequently transferred electrophoretically onto nitrocellulose membranes (Amersham Pharmacia, UK), followed by an hour-long incubation at 4°C in a 5% milk TBST solution to block non-specific binding. Primary antibodies specific to *GHR* (1:500 dilution, no. 20713-1-AP, Proteintech, China) were then used to probe the liver protein. After a 1-hour incubation with the secondary antibody and washing in TBST, the membranes were subjected to chemiluminescence imaging using a Bio-Red ChemiDoc XRS system (U.S.) to visualize the protein bands.

### 2.10 Transcriptomics assay

Fresh livers from 6-month-old *GHR* ^−/−^ pigs as well as wild-type pigs were stored at −80°C and transported using dry ice to be sent to the company of Shanghai Zhong ke New Life for transcriptomics analysis.

### 2.11 Statistical analysis

SPSS statistical software (version 17.0) was used for all data analysis, while GraphPad Prism was used for data organization (La Jolla, CA). The data sets from the two groups were compared using Dunnett’s t-test. *p* < 0.05 was deemed significant.

## 3 Results

### 3.1 *In vitro* transfection of specific rAAV serotypes in porcine zygotes

**FIGURE 1:**
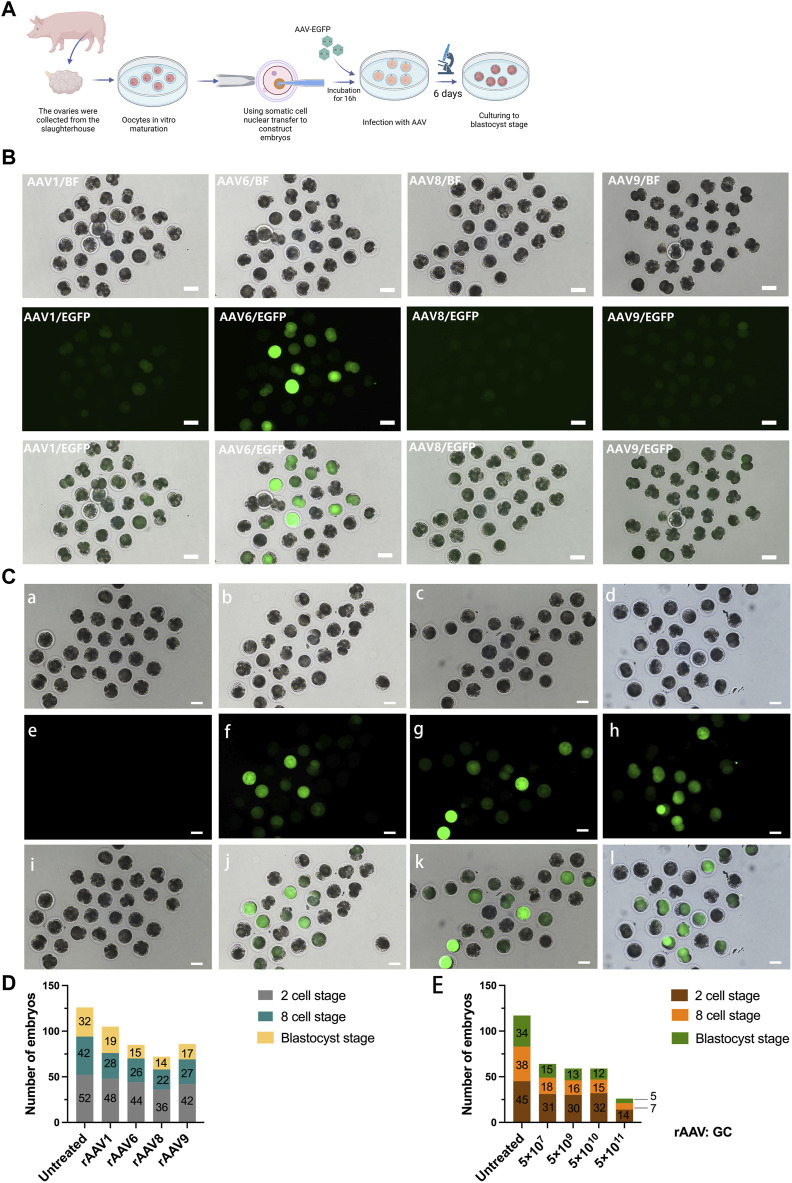
Transduction of Preimplantation Pig Embryos using rAAV Vectors. **(A)**: Schematic representation of the experimental workflow, encompassing embryo preparation, embryo infection, and subsequent *ex vivo* assessment. **(B)**: Evaluation of multiple rAAV serotypes for their transduction efficiency in pig embryos *ex vivo*. Scale bars, 100 μm. **(C)**: Microscopic images of pig embryos infected with scAAV6-CMV-EGFP vectors at four distinct doses. Scale bars, 100 μm. a, e: 5 × 10^7^ GC; b, f: 5 × 10^9^ GC; c, g: 5 × 10^10^ GC; d, h: 5 × 10^11^ GC. **(D)**: Stacked bar graphs of the number of porcine embryos at different developmental periods after transfection with different rAAV serotypes at the 2-cell, 8-cell, and blastocyst stages, a total of 80 embryos were used in each group. **(E)**: Stacked bar graphs of the number of porcine embryos at different developmental periods after transfection with varying doses of scAAV6-CMV-EGFP at the 2-cell, 8-cell, and blastocyst stages, a total of 70 embryos were used in each group.

Building on prior research, we conducted an assessment of four rAAV serotypes and incubated porcine fertilized eggs with rAAV1, rAAV6, rAAV8, and rAAV9, all carrying enhanced green fluorescent protein (EGFP) at a dose of 5 × 10^9^ genome copies (GCs) over a 16-hour period (Figure 1A). The embryos were cultured and observed continuously for 6 days after transfection until they developed to the blastocyst stage, and the developmental and transfection effects of the embryos in each group were compared during the period. During the initial 24-hour culture, no discernible fluorescence expression was observed in pig embryos treated with the four rAAV serotypes at the same dose. However, after incubation from 36 h to 72 h, fluorescent expression was detected in all four groups of embryos. In the untreated group, no fluorescent signals were observed (Supplementary Figure S1). Notably, embryos co-incubated with serotype 6 rAAV exhibited significantly stronger fluorescence intensity compared to the other groups, followed by serotype 1 rAAV, while fluorescence expression remained faint in embryos incubated with serotype 8 and serotype 9 rAAV (Figure 1B). These findings emphasize the superior effectiveness of serotype 6 rAAV for porcine fertilized eggs.

Additionally, we examined the effects of rAAV transfection on embryo development. The findings showed that the untreated control group had a blastocyst development rate of about 40% (32/80, 80 embryos total); in the rAAV-transfected group, rAAV1 was 23.8% (19/80), rAAV 6 was 18.8% (15/80), rAAV 8 was 17.5% (14/80), and rAAV 9 was 21.3% (17/80). These findings suggest that rAAV incubation exerts some influence on embryonic development (Figure 1D).

We proceeded to explore the optimal transfection dose for achieving maximum transfection efficiency while preserving embryo development rates. To assess the effects of embryo transfection and embryo development, we employed scAAV6-CMV-EGFP with four different doses (5 × 10^7^ GC, 5 × 10^9^ GC, 5 × 10^10^ GC, and 5 × 10^11^ GC). Our findings revealed that, after 72 h of transfection, the 5 × 10^7^ GC dosage exhibited negligible fluorescence expression, while the 5 × 10^9^ GC, 5 × 10^10^ GC, and 5 × 10^11^ GC dosages exhibited robust fluorescence expression (Figure 1C).

The embryo development rates for embryos transfected with 5 × 10^7^ GC, 5 × 10^9^ GC, and 5 × 10^10^ GC ranged from 17.1% to 21.4% (Figure 1E, a total of 70 embryos in each group), while embryos transfected with 5 × 10^11^ GC exhibited a significantly lower development rate of only 7% (5/70). Consequently, to attain higher transfection efficiency and gene editing effectiveness while minimizing adverse effects on embryo development, we selected the 5 × 10^10^ GC dosage for testing the delivery of CRISPR/Cas9 by rAAV for *in vivo* and *in vitro* embryonic gene editing.

### 3.2 *In vitro* gene editing through transfection of pig-fertilized eggs using rAAV

**FIGURE 2:**
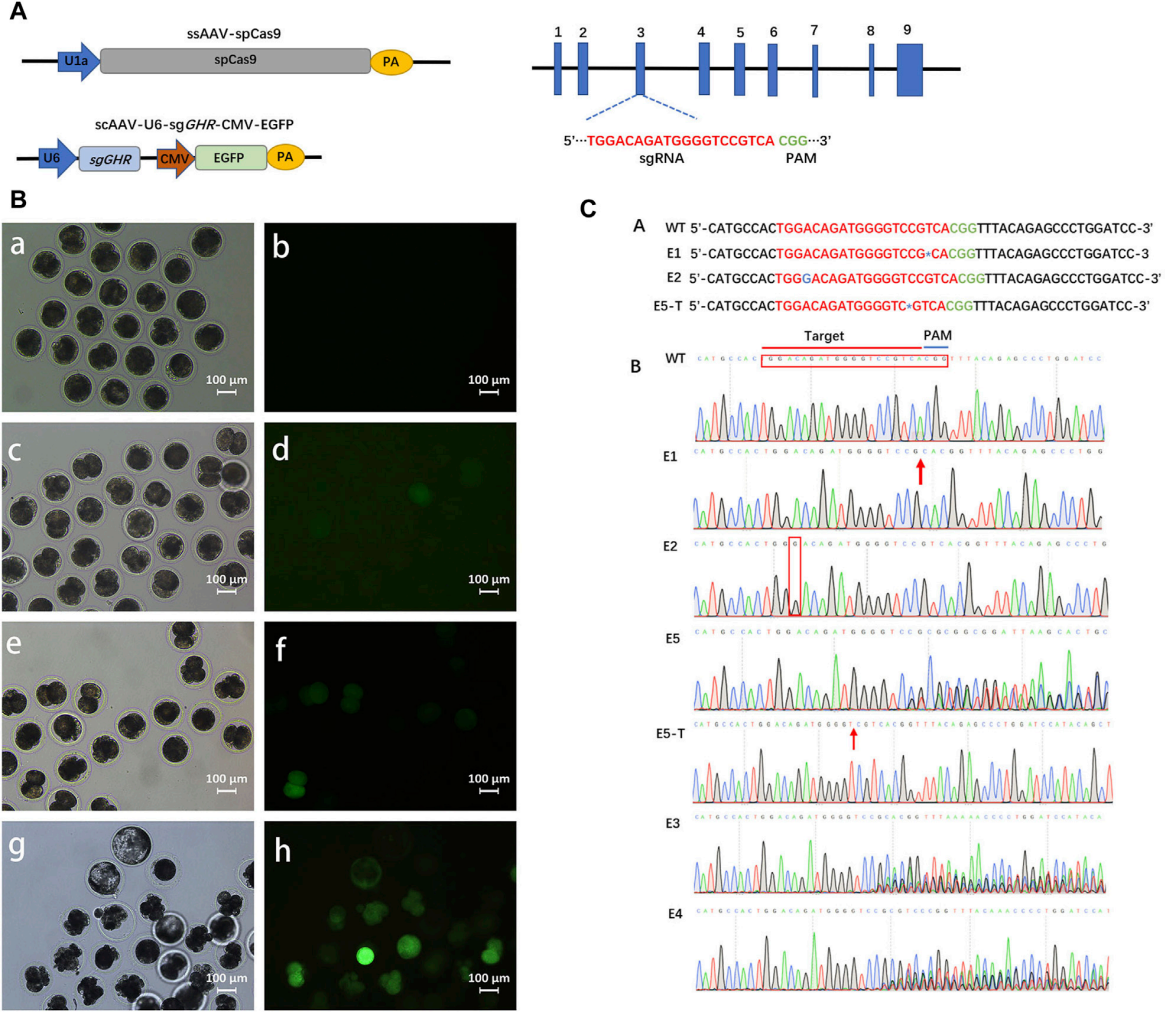
Gene editing in intact pre-implantation embryos transduced with rAAV vectors. **(A)**: Schematics of the constructions expressing SpCas9 and sgRNA, respectively. **(B)**: White light field of view as well as fluorescence field of view after 24 h , (a, b), 36 h , (c, d), and 72 h, (e, f) of fertilized eggs in pigs transfected with rAAV targeting the *GHR* gene; g, h: Developed blastocysts are visible at day 6. Scale bars, 100 μm. **(C)**: *In vitro* embryonic gene editing sequencing.

The preceding experiments have provided compelling evidence of rAAV’s ability to traverse the zona pellucida in pigs. In order to assess the efficacy of rAAV-CRISPR/Cas9-mediated *in vitro* embryonic gene editing, we formulated three single guide RNAs (sgRNAs) designed to target exon 3 of the *GHR* gene (Figure 2A). The sgRNA targeting efficiency was evaluated within pig kidney fibroblasts. The outcomes of the TIDE analysis revealed that sgRNA #1 exhibited the highest targeting efficiency at 79.7%, followed by sgRNA #2 at 66.7%, and sgRNA #3 at 72.9%. Consequently, sgRNA #1 was selected for packaging into the rAAV6 vector for *GHR* gene targeting (Supplementary Figure S2).

Following this, the zygotes were subjected to incubation with a 1:1 blend of scAAV6-U6-sg*GHR*-CMV-EGFP and ssAAV6-U1a-spCas9 at a dosage of 5 × 10^10^ GC for 16 h. Subsequently, the culture medium was replaced with fresh medium, and the zygotes were allowed to develop to the blastocyst stage. Notably, fluorescence expression within the embryos remained inconspicuous at the 24-hour mark post-transfection. However, a discernible increase in fluorescence was observed commencing from the 36-hour time point (Figure 2B). In the subsequent step, genomic DNA was extracted from the embryos and subjected to sequencing for target site analysis.

A total of twenty embryos successfully progressed to the blastocyst stage, all of which were subjected to targeted gene sequencing analysis. The sequencing data confirmed the effective delivery of the CRISPR/Cas9 system to porcine embryos via rAAV, resulting in the introduction of genetic mutations. Among these, two embryos exhibited biallelic mutations (Figure 2C). Specifically, embryo #1 (E1) manifested a biallelic mutation characterized by a T-base deletion proximal to the PAM sequence, whereas embryo #2 (E2) displayed a biallelic mutation marked by a G-base insertion. Notably, embryo #5 (E5) demonstrated a heterozygous mutation, featuring a C-base deletion on one allele near the PAM sequence, as substantiated by T-vector sequencing. Moreover, sequencing analysis of three embryos at the target site revealed multiple sets of peaks, indicative of diverse mutations and chimerism (Figure 2C). Remarkably, the remaining fourteen embryos exhibited an absence of mutations. In the context of *in vitro* transfection experiments, the mutation rate was ascertained to be 30% (6 out of 20 embryos), with a biallelic mutation rate of 10% (2 out of 20 embryos). These findings underscore the capability of rAAV6 to effectively deliver the CRISPR/Cas9 system to porcine fertilized eggs, enabling *in vitro* gene editing.

### 3.3 *In vivo* delivery of rAAV-CRISPR/Cas9 to produce *GHR* gene-edited pigs

**FIGURE 3:**
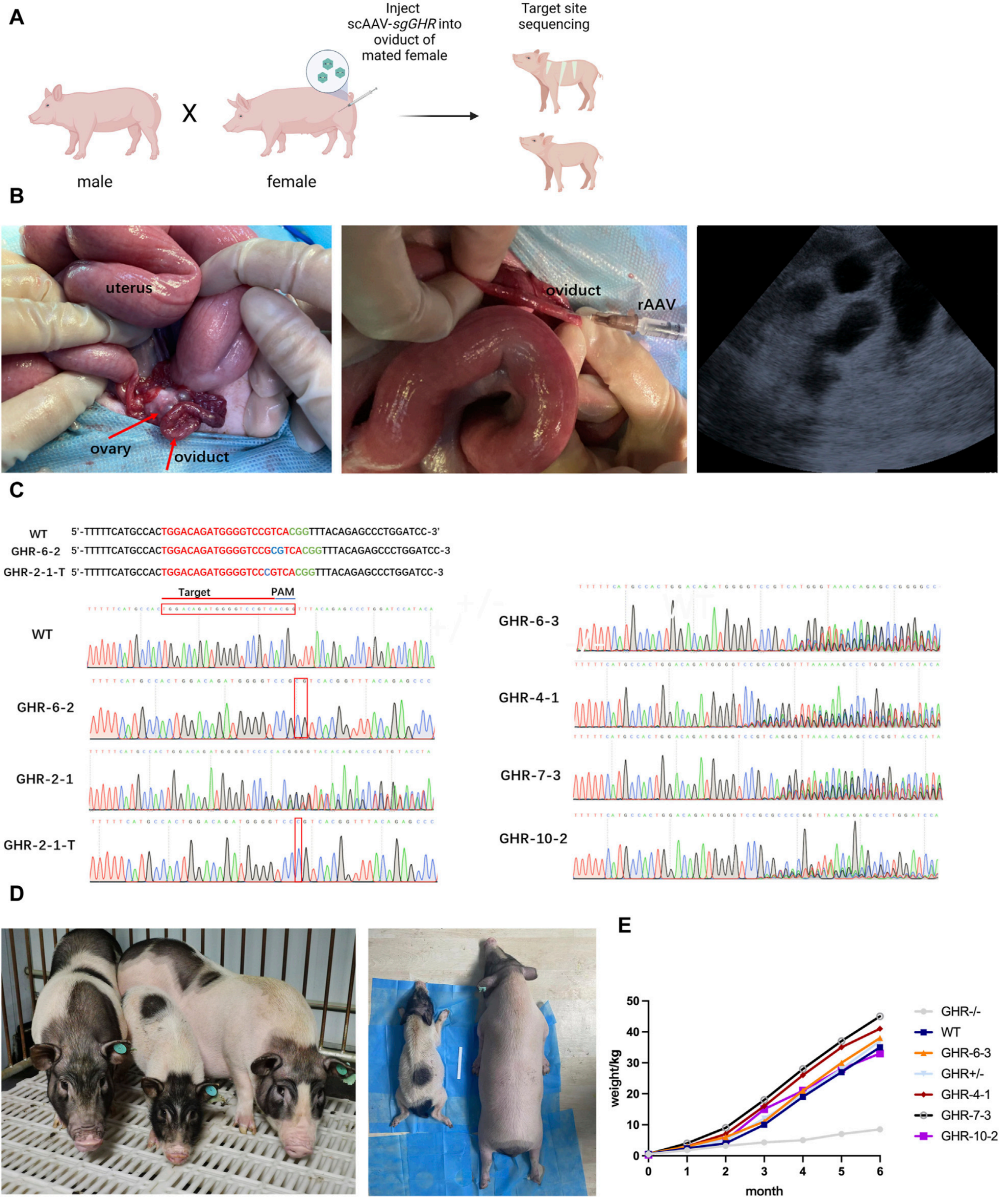
Generating and observing *GHR* gene-edited pigs. **(A)**: Flowchart of the rAAV oviductal injection method for obtaining gene-edited pigs in one step. **(B)**: Oviduct injection surgery and ultrasound testing. From left to right: Porcine ovary and fallopian tube structures; injected with rAAV-CRISPR/Cas9, and ultrasound results showing the gestational sac 30 days after surgery. **(C)**: Sequencing results of gene-edited pig fetuses produced by *in vivo* injection method; **(D)**: The left graph compares the size of *GHR* ^−/−^ pigs with that of WT and *GHR* ^+/−^ pigs of the same age; the right graph compares *GHR* ^−/−^ pigs under anesthesia with that of wild-type pigs, and the difference in size can be seen visually. **(E)**: Weight gain curves (1–6 months of age body weight) of *GHR* ^−/−^ pigs, WT, *GHR* ^*+/−*^, and four pigs with multiple peaks in the ear tissue.

To corroborate the potential for *in vivo* pre-implantation embryo gene modification by the introduction of viral particles into the oviduct of pregnant females, we administered a 1:1 blend of ssAAV6-U1a-spCas9 and scAAV6-U6-sg*GHR*-CMV-EGFP (at 5 × 10^10^ GC) into the ampulla of the oviduct, where fertilization takes place, within 24 h after conception in sows (Figure 3B). A total of 11 pigs were subjected to the rAAV targeting the *GHR* gene, and pregnancy was ascertained through ultrasound examination 30 days post-injection (Figure 3B). Among these, 6 sows were confirmed to be pregnant, resulting in a pregnancy rate of 54.5%.

At approximately 114 days of gestation, a total of 26 piglets were successfully delivered, and genotyping of the piglets’ ears was conducted to identify the initial target loci. The genotyping outcomes unveiled that one pig (piglet *GHR*-6-2) exhibited biallelic mutation, characterized by a 2-base insertion near the PAM sequence. Another pig (piglet *GHR*-2-1) exhibited two sets of peaks in the sequencing data, signifying heterozygous mutation with a one-base insertion in one of the sequences following T-vector cloning (Figure 3C for *GHR*-2-1-T). The sequencing results of four additional piglets displayed multiple sets of peaks near the target site, suggesting the presence of multiple mutations, while the remaining piglets were determined to be of the wild-type genotype. The overall mutation rate was calculated to be 23.08%, with a biallelic mutation rate of 3.8%. Comprehensive sequencing data are presented in Figure 3C.

At 6 months of age, organ sampling was conducted on the pigs carrying the mutations, and DNA extraction was performed for sequencing of the target loci. The sequencing results from different organs of the *GHR*-6-2 biallelic mutant piglets and the *GHR*-2-1 heterozygous mutant pigs were consistent with the ear sequencing results.

To assess the growth patterns of the six pigs bearing genetic mutations, we diligently monitored their weight changes over 6 months. As these pigs underwent maturation, conspicuous differences in body size became evident, distinguishing the *GHR* biallelic mutant pigs from their wild-type counterparts. In stark contrast, pigs with heterozygous mutations or those exhibiting multiple peaks displayed no substantial deviations in body size in comparison to the wild-type pigs (Figure 3D).

By the end of the 6-month observation period, the *GHR* biallelic mutant pigs exhibited a weight of 8.5 kg, whereas the heterozygous mutant pigs weighed 37 kg, the wild-type pigs weighed 35 kg, and the other four pigs with multiple peaks in their ears had respective weights of 38 kg (*GHR*-6-3), 41 kg (*GHR*-4-1), 45 kg (*GHR*-7-3), and 33 kg (*GHR-*10-2) (as depicted in Figure 3E).

### 3.4 Biochemical features of *GHR* gene-edited pigs

**FIGURE 4:**
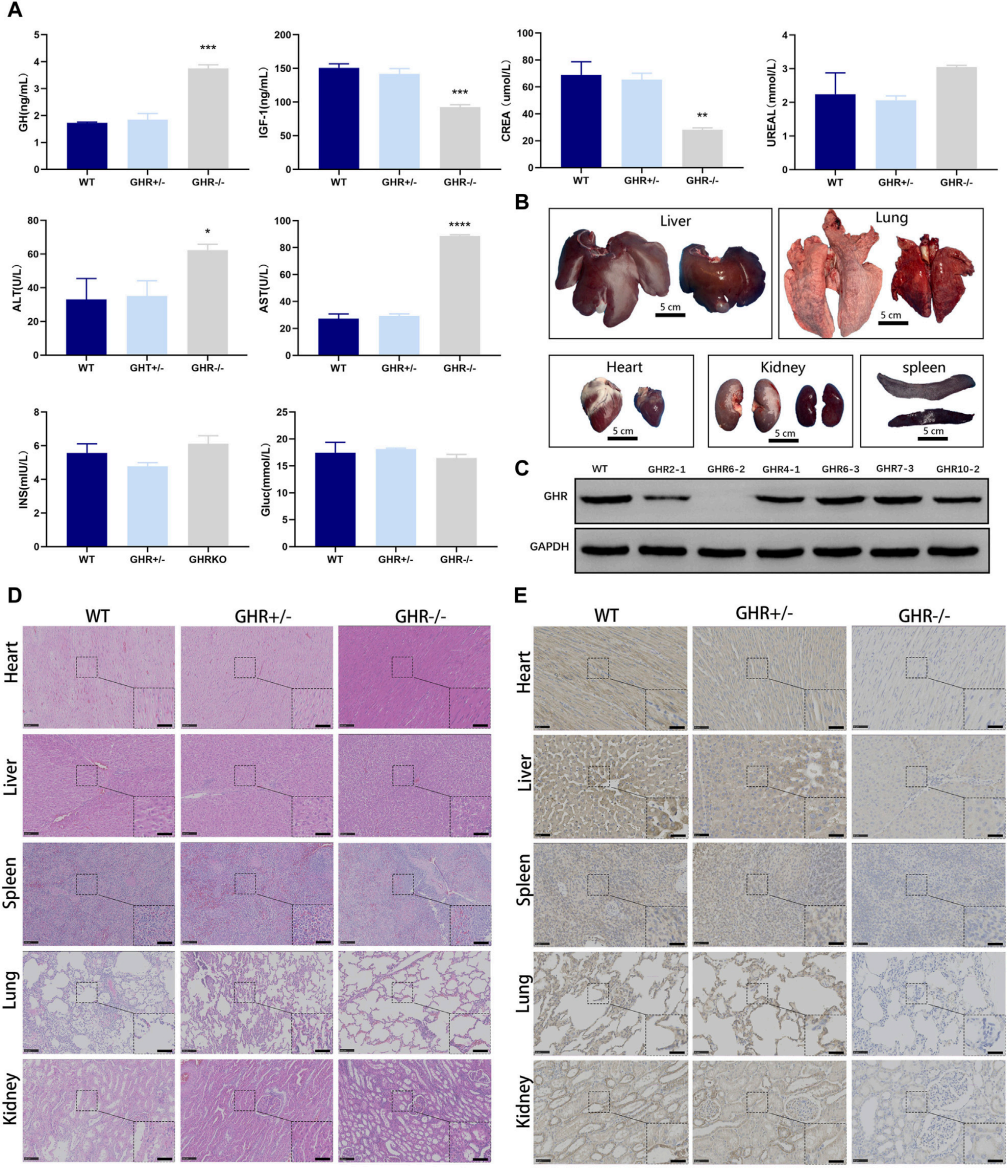
Phenotypic characterization of gene-edited pigs produced by *in vivo* injection of rAAV-CRISPR/Cas9. **(A)**: Serum biochemical assay in *GHR* gene-edited pigs. *p < 0.001, (n = 3). **(B)**: Organ growth in *GHR*-KO compared with WT pigs. Scale bar, 5 cm. **(C)**: WB results of liver tissue from GHR^−/−^ pigs with WT, GHR^+/−^ pigs, and four pigs with stray peaks in ear tissue. **(D)**: H&E and **(E)**: IHC of *GHR* gene pig-edited pigs compared with WT pigs. Left scale bar, 100 μm. Right scale bar, 50 μm.

Simultaneously, to comprehensively assess the physiological alterations induced by *GHR* knockout, we conducted biochemical assays on sera obtained from 6-month-old wild-type, *GHR* ^*−/−*^, and *GHR* ^*+/-*^ pigs. Serum levels of growth hormone (GH) were notably elevated in *GHR* ^−/−^ pigs in comparison to wild-type and heterozygous mutant pigs, indicative of a disruption in the negative feedback regulation of GH (Figure 4A). Additionally, we evaluated the levels of Insulin-Like Growth Factor 1 (IGF1), a growth-promoting factor governed by GH, in the pig serum.

Remarkably, *GHR* ^−/−^ pigs displayed significantly reduced levels of IGF1 in contrast to their wild-type counterparts, while no substantial disparity was observed between *GHR* ^*+/−*^ pigs and wild-type pigs (Figure 4A). These findings lend support to the notion that single-allele mutations in *GHR* do not elicit discernible phenotypic alterations, while biallelic mutations are responsible for the observed reductions in body size among pigs.

Biochemical tests were conducted to investigate the physiological effects of *GHR* mutation in pigs. At 6 months of age, *GHR* ^−/−^ pigs exhibited elevated serum levels of alanine transaminase (ALT) and glutamic oxalacetic transaminase (AST) when compared to heterozygous mutants and wild-type pigs. Conversely, there were no significant differences in these liver function-related indicators between *GHR* ^*+/−*^ and wild-type pigs.

Regarding glucose metabolism, as measured by insulin (INS) and glucose (Gluc) levels, there were no significant differences observed between *GHR* gene mutants and wild-type pigs (Figure 4A). The assessment of kidney function, as indicated by urea (Urea) levels, also showed no significant variations between *GHR* ^−/−^ and wild-type pigs. *GHR* ^−/−^ pigs displayed a lean phenotype compared to their wild-type counterparts and had notably lower serum creatinine (CREA) levels (Figure 4A).

Furthermore, an analysis of lipid-related indicators revealed significantly reduced cholesterol (CHO2) levels in *GHR* ^*−/−*^ pigs in comparison to wild-type and *GHR* ^+/−^ pigs, which may be linked to abnormal liver metabolism. However, no significant differences in triglyceride (TRIGL) levels were observed among the groups (Figure 4A).

### 3.5 Morphology and pathology of *GHR* gene-edited pigs

As depicted in Figure 4B, the organs of 6-month-old GHR^−/−^ pigs exhibited noticeable differences in size when compared to wild-type pigs, displaying organ sizes consistent with their body proportions. The Western blot analysis of liver samples obtained from the six-month-old pigs further affirmed the absence of GHR protein expression in GHR^−/−^ pigs (Figure 4C). In order to further investigate the pathological morphological changes in *GHR* ^−/−^ pigs, we obtained tissue samples from various organs of 6-month-old pigs and conducted H&E staining, comparing them to age-matched wild-type pigs.

Growth hormone has a broad spectrum of action, affecting nearly all tissues. Therefore, to examine whether *GHR* gene knockout had a direct impact on the microstructural organization of various organs (heart, liver, spleen, lung, and kidney), we conducted H&E staining on tissue sections from these organs. The results indicated that, in comparison to wild-type pigs, there were no significant morphological abnormalities in the tissues of these organs in *GHR* ^*−/−*^ pigs (Figure 4D). Subsequently, we performed immunohistochemical validation of GHR protein expression in these organs. The results revealed that wild-type pigs and heterozygous mutant pigs displayed *GHR* protein expression in the mentioned organs, while *GHR* ^*−/−*^ pigs exhibited an absence of *GHR* protein (Figure 4E).

### 3.6 Transcriptomic analysis of *GHR−/−* pig

**FIGURE 5:**
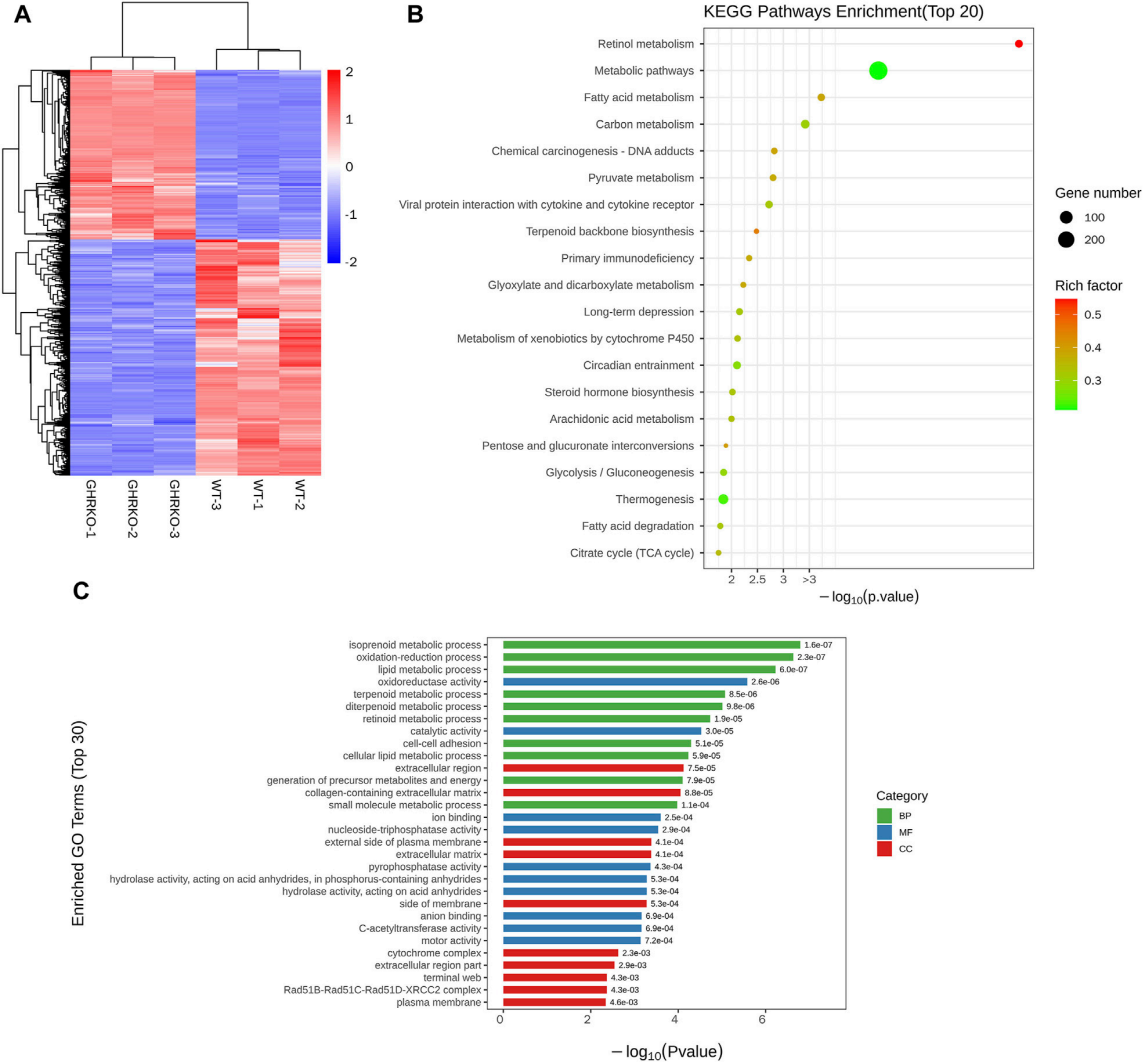
Transcriptomic analysis of *GHR*KO pigs. **(A)**: Differential gene cluster map. **(B)**: KEGG enriched bubble map of differentially expressed genes. **(C)**: Differential gene GO enrichment histogram.

To further explore the effects resulting from *GHR* knockdown, identify potential underlying pathways responsible for these effects, and lay the groundwork for clinical animal disease modeling, we conducted transcriptomic sequencing analysis on *GHR* ^−/−^ porcine livers. The clustering of differentially expressed genes between the *GHR* knockout (*GHR*KO) group and the wild-type is illustrated in Figure 5A. Differential gene KEGG enrichment analysis revealed predominant impacts on metabolic pathways, fatty acid metabolism, carbon metabolism, and glycolysis/gluconeogenesis Figure 5B. Additionally, differential GO functional enrichment analysis indicated effects on processes such as lipid metabolism, oxidation-reduction, and isoprenoid metabolic processes (linked to cholesterol synthesis, Figure 5C). These findings provide valuable insights into the molecular mechanisms associated with *GHR* knockdown-induced alterations, offering a foundation for future clinical animal disease modeling endeavors.

## 4 Discussion

Our study is the first time that rAAV-based vectors have been used to transduce intact pre-implantation embryos in either *ex vivo* or *in vivo* in pig. Compared to the lentiviral vectors that necessitate the removal of the zona pellucida or microinjection into the perivitelline space, this rAAV-CRISPR/Cas9-mediated gene delivery method offers a distinct advantage by obviating the need for zona pellucida removal (Lois et al., 2002; Pfeifer et al., 2002). Furthermore, the generation of genetically modified pigs is made possible by the use of rAAV vectors rather than methods that call for sophisticated equipment, including pronuclear injection.

Adeno-associated viruses (AAVs) are known for their characteristic tissue and cell specificity. Prior research has illuminated this specificity, illustrating that rAAV types 1 and 6 exhibit remarkable targeting precision in mouse and rat embryos, while rAAV type 6 demonstrates a similar proficiency in monkey embryos (Mizuno et al., 2018; Yoon et al., 2018; Wang et al., 2019b). In the quest to identify the rAAV serotype most adept at homing in on porcine zygotes, we initiated preliminary *in vitro* assessments. These experiments involved transfecting porcine embryos with rAAV types 1, 6, 8, and 9, all encoding EGFP, to assess their transduction efficiency and identify the serotype that delivered the highest efficacy.

The results showed that all four serotypes were capable of transducing porcine zygotes, but rAAV6 exhibited significantly stronger transduction than other serotypes. Therefore, we chose to use rAAV6 to package the CRISPR/Cas9 system for *GHR* gene targeting.

Commonly employed recombinant adeno-associated viruses (rAAVs) include two main variants: single-stranded adeno-associated virus (ssAAV) and self-complementary adeno-associated virus (scAAV) (Fisher et al., 1996). After entering the host, ssAAV requires a transcription process, which is a rate-limiting step affecting transduction efficiency (Ferrari et al., 1996). scAAV forms a double-stranded structure by circularization between the molecules in the inverted terminal repeat (ITR) region. Compared to traditional ssAAV, scAAV has a more stable structure and can express the packaged gene more quickly and efficiently (Ferrari et al., 1996). However, due to the formation of circular structures, scAAV has lower packaging capacity than ssAAV (Drouin et al., 2016).

Considering the finite packaging capacity of rAAV, which is approximately 4.8 kilobases (kb) (Wang et al., 2019a), and the standard size of the commonly used spCas9 fragment (∼4.2 kb) (Singh et al., 2018), packaging both CRISPR/Cas9 and sgRNA within the same vector poses a challenge. Typically, a dual-vector system is used to package the CRISPR system (Wang et al., 2020). Additionally, studies have shown that the therapeutic effect of CRISPR/Cas9 mediated by scAAV in treating Duchenne muscular dystrophy (DMD) in rats is at least 20 times higher than that of ssAAV-mediated CRISPR/Cas9 (Zhang et al., 2020). In line with the goal of enhancing the editing efficiency of CRISPR/Cas9 and mitigating the occurrence of embryo chimerism, we adopted a strategy of packaging sgRNA-*GHR* within scAAV and spCas9 within ssAAV.

In this study, we successfully achieved porcine embryo *GHR* gene editing through *in vitro* embryo transfection experiments. The *in vitro* embryo gene editing rate reached 35%, with a homozygous editing rate of 10%, but chimerism occurred. In comparison to the gene editing efficiency achieved through rAAV transduction in mouse embryos (80%–100%), our data may appear relatively modest. However, several factors contribute to this discrepancy, including the inability to precisely control the amount of CRISPR-Cas9 components reaching the embryos, sgRNA efficiency, and the selection of target gene loci (Chen et al., 2016; Mizuno et al., 2018; Yoon et al., 2018).

It seems that mosaicism in individual CRISPR/Cas9-edited founder animals is a regular occurrence in genome editing techniques (Yang et al., 2014; Huai et al., 2017), like ours. Multiple studies have shown that the occurrence of chimeric animals is caused by multiple factors, such as the delivery method of the CRISPR/Cas9 system, the timing of CRISPR/Cas9 system entry into the embryo, sgRNA selection, and target site microenvironment (Yang et al., 2013; Yen et al., 2014).

Moreover, given the constrained packaging capacity of rAAV, it is a common practice to employ two separate vectors for spCas9 and sgRNA. However, this approach cannot ensure the precise ratio and synchronized entry of both vectors into the embryos. Consequently, it may impact the efficiency of CRISPR/Cas9 action during early embryo development and contribute to the occurrence of chimerism. Notably, an alternative approach warrants exploration. *Staphylococcus* aureus-derived saCas9, with a smaller size of only 3.16 kb (Ran et al., 2015), could potentially offer a strategy for reducing chimerism rates by packaging both sgRNA and saCas9 within a single vector.

Following our successful exploration of *in vitro* transfection for porcine embryo gene editing using rAAV, we ventured into investigating the potential of *in vivo* transfection through oviductal injection of adeno-associated viruses. In this study, we conducted oviductal virus injections targeting the *GHR* gene with rAAV-CRISPR/Cas9 in 11 fertile sows. The outcomes were promising, with 6 sows achieving pregnancy and ultimately delivering a total of 26 piglets. Among these piglets, one displayed biallelic mutations, one exhibited a heterozygous mutation, four presented chimeric ear tissues, and the remainder were of the wild-type genotype.

The relatively low pregnancy rate among the sows could be attributed to surgical stress and potential conception failure, as natural mating was employed for conception. To address this concern, alternative strategies such as artificial insemination or the co-injection of semen with rAAV-CRISPR/Cas9 into the oviduct can be considered in subsequent studies. These approaches could offer precise control over the timing of conception and, importantly, enable more accurate control over the timing of CRISPR/Cas9 embryo transfection. This, in turn, holds the potential to reduce embryo chimerism rates and further refine the gene editing process.

We conducted a comprehensive assessment of *GHR* gene-edited pigs over a 6-month period until they reached sexual maturity. Notably, starting at the age of 3 months, *GHR* ^−/−^ pigs exhibited a progressive and significant divergence in body weight compared to their wild-type counterparts. This distinction persisted and became more pronounced as they reached sexual maturity at 6 months.

Conversely, neither the heterozygous mutant pigs nor the four pigs with chimeric attributes displayed any statistically significant differences in body size when compared to the wild-type pigs.

Biochemical assays further corroborated our observations, affirming that *GHR* ^*−/−*^ pigs exhibited markedly elevated levels of growth hormone (GH) and significantly reduced levels of insulin-like growth factor 1 (IGF1) in comparison to the wild-type pigs. These outcomes closely aligned with the expected phenotypic alterations, providing robust evidence of the successful modeling and the method’s feasibility. In contrast, none of the heterozygous mutant pig exhibited statistically significant distinctions from the wild-type counterparts.

Subsequently, we conducted organ tissue sampling on 6-month-old *GHR* ^*−/−*^ pigs to further assess the impact of *GHR* knockout. These pigs exhibited smaller organs proportionate to their reduced body size. Histological examination, including H&E staining, revealed no significant differences in organ tissue histomorphology when compared to wild-type pigs.

Immunohistochemical and Western blot analyses confirmed the absence of *GHR* protein expression in *GHR* ^*−/−*^ pigs. Additionally, serum biochemical assays revealed elevated levels of ALT and AST in liver function assessments, as well as increased urea levels in kidney function assessments. However, there were no significant deviations in blood glucose and insulin levels. These findings align with previously reported symptoms in the literature, affirming the method’s effectiveness in generating animal models (Hinrichs et al., 2018; Riedel et al., 2020). Furthermore, in pursuit of a more comprehensive understanding of *GHR* gene-deficient pigs, we conducted transcriptomic sequencing on *GHR* ^*−/−*^ pig and WT pig. The results unveiled significant distinctions between *GHR* ^*−/−*^ pigs and their wild-type counterparts, with notable enrichment observed in metabolic pathways, including those related to fatty acid metabolism, carbon metabolism, lipid metabolism, cholesterol metabolism, and other pertinent pathways. This initial exploration lays the groundwork for future investigations into the potential application of *GHR* ^*−/−*^ pigs as xenograft donors and their utility in developing clinical animal disease models.

The aforementioned findings highlight the successful application of gene editing in porcine embryos, both *in vitro* and *in vivo*, employing rAAV6-CRISPR/Cas9. It is important to note that the current likelihood of obtaining healthy gene-edited piglets through somatic cell nuclear transfer techniques typically falls within a range of 1%–5% (Gouveia et al., 2020), and our probability of obtaining biallelic gene-edited pigs by rAAV-mediated CRISPR/Cas9 tubal injection strategy was 3.8% (1/26). While this study necessitates a larger number of conceived sows for tubal injection and confines itself to evaluating the feasibility of single knockouts, its notable contribution lies in the substantial streamlining of gene-edited pig production. By eliminating the *in vitro* gene editing step and bypassing the complex procedures involved in embryo microinjection and embryo transfers, it presents a promising pathway for enhancing the efficiency and practicability of gene editing within the realm of pig breeding.

The results of this study can be widely applied not only to the production of other animal models and provide a facile way for gene editing animal model preparation but also as a strategy for *in vivo* gene therapy at the animal embryo level. Gene therapy at the embryonic level is achieved by injecting rAAV carrying the CRISPR system or the base editor system into the oviduct to genetically correct the embryo. In the next study, we will further test different delivery vectors and different CRISPR systems, and also improve the delivery time and dose of CRISPR systems, aiming to improve the efficiency of biallelic mutations in embryos and further refine the effectiveness of the method.

## Data Availability

The datasets presented in this study can be found in online repositories. The names of the repository/repositories and accession number(s) can be found in the article/Supplementary Material.
