# Supplementary material for: One-step in vivo gene knock-out in porcine embryos using recombinant adeno-associated viruses
Source: Front Cell Dev Biol. 2024 Mar 15;12:1376936. doi: 10.3389/fcell.2024.1376936 (PMC10978582; doi:10.3389/fcell.2024.1376936)
Supplement: Supplementary file 1 [file DataSheet1.docx]

Supplementary Material

One-step in vivo gene knock-out in porcine embryos using recombinant adeno-associated viruses

Mengyu Gao^1^*, YuTing He^1^, XingLong Zhu^1^, WanLiu Peng^1^, YanYan Zhou^1^, Yang Deng^1^, Guangneng Liao^2^, Wei Ni^3^, Yi, Li^4^, Jun Gao^5^, Hong Bu^1^, JiaYin Yang^6^, Guang Yang^2^, Yang Yang^7^, JiBao^1*^

*** Correspondence:** Corresponding Author: JiBao， [baoji@scu.edu.cn](mailto:baoji@scu.edu.cn).

# Supplementary Figures

## Supplementary Figure1


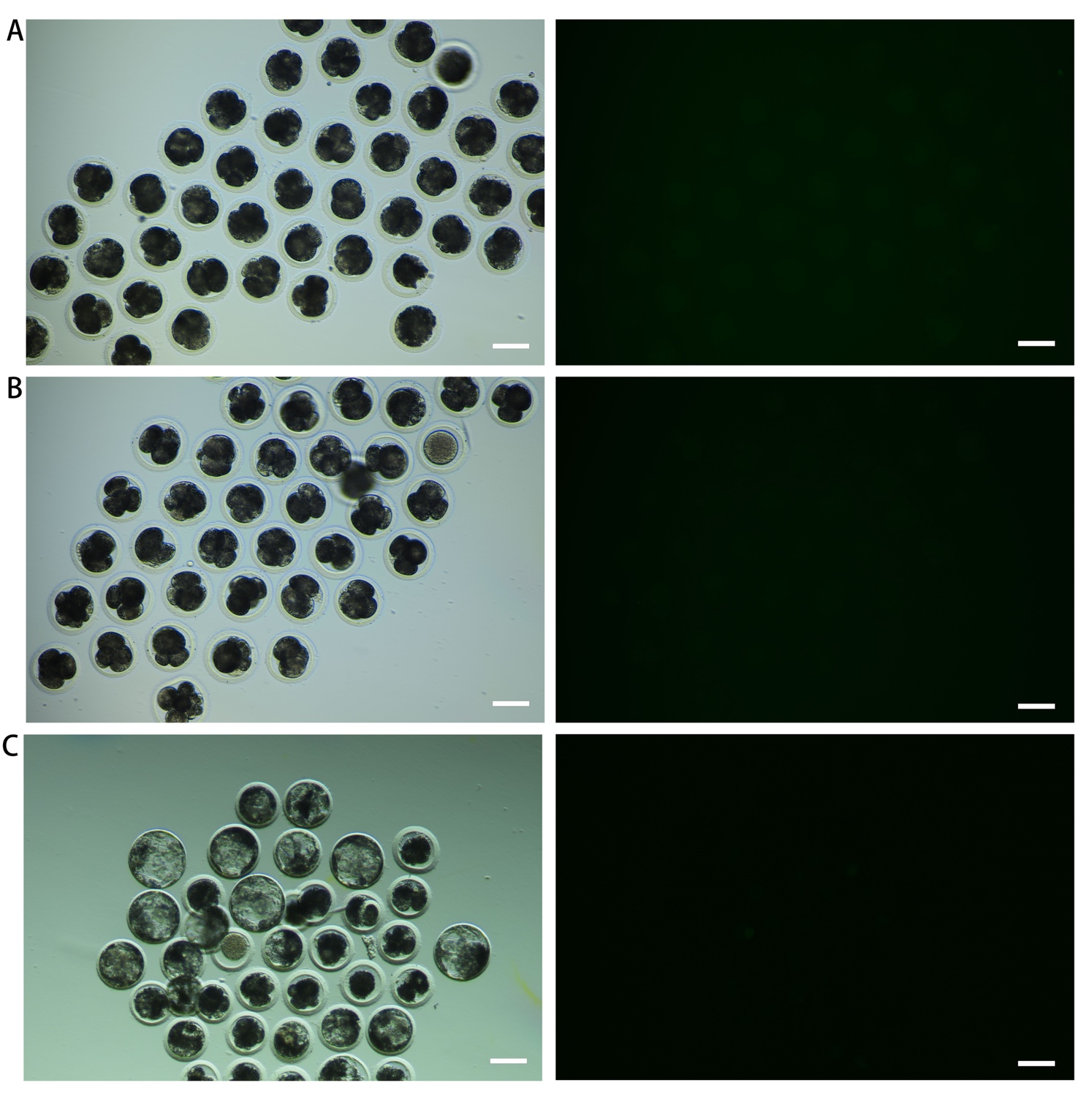


**Supplementary Figure 1.** Cultivation of pig embryos to blastocysts as contral. White light and fluorescent fields of embryos without rAAV transfection cultured for 48h(A),72h(B) and blastocysts(C). scale bar, 100 μm.

## Supplementary Figure 2


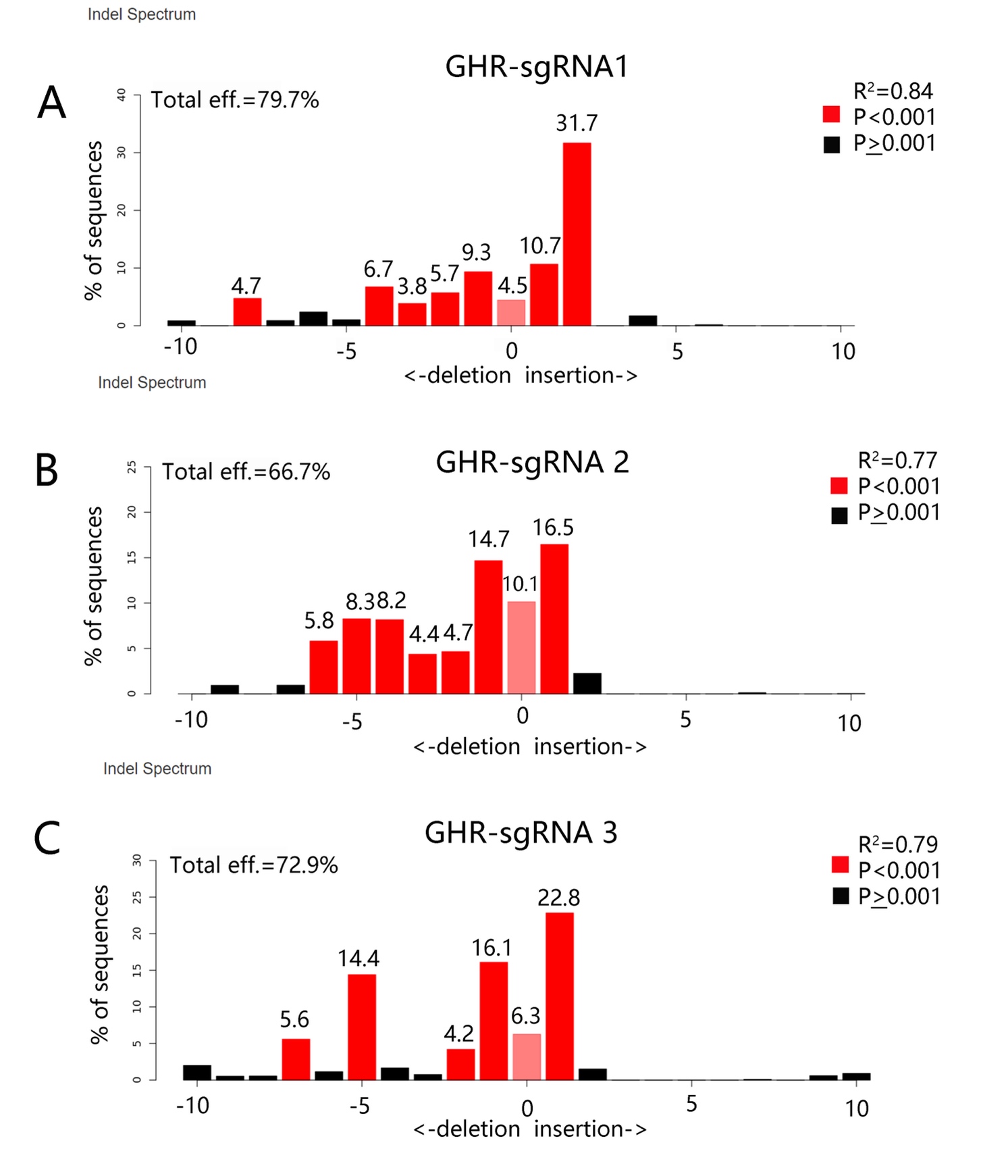


**Supplementary Figure 2.** Analysis of sgRNA efficiency targeting the GHR gene by TIDE. sgRNA #1 exhibited the highest targeting efficiency at 79.7%(A), followed by sgRNA #2(B) at 66.7%, and sgRNA #3 at 72.9%(C).
